# 2-Ferrocenyl-2-[(2-ferrocenylethen­yl)(morpholin-4-yl)meth­yl]-1,3-di­thiol­ane

**DOI:** 10.1107/S2414314624002347

**Published:** 2024-03-21

**Authors:** Claudia Oliva-Colunga, Jessica J. Sánchez García, Marcos Flores-Alamo, Elena I. Klimova

**Affiliations:** a Facultad de Química, Universidad Nacional Autónoma de México, Ciudad Universitaria, Ciudad de México, 04510, Mexico; Sunway University, Malaysia

**Keywords:** crystal structure, morpholine, ferrocen­yl, stereochemistry

## Abstract

The mol­ecular structure of 2-ferrocenyl-2-[(2-ferrocenylethen­yl)(morpholin-4-yl)meth­yl]-1,3-di­thiol­ane, [Fe_2_(C_5_H_5_)_2_(C_19_H_21_NOS_2_)] or C_29_H_31_Fe_2_NOS_2_, has the ferrocenyl fragments in a *trans* disposition with respect to the vinyl group. One of the methyl­ene groups is disordered over two sites with occupancies of 0.782 (13):0.218 (13). In the crystal, cyclo­penta­dienyl-C—H⋯O(morpholin­yl) inter­actions feature within helical chains parallel to the *c*-axis direction. The chains are connected by methyl­ene- and cyclo­penta­dienyl-C—H⋯O(cyclo­penta­dien­yl) inter­actions.

## Abstract 2

The structure of 2-ferrocenyl-2-[(2-ferrocenylethen­yl)(morpholin-4-yl)meth­yl]-1,3-di­thiol­ane, is described. In the crystal, cyclo­penta­dienyl-C—H⋯O(morpholin­yl) inter­actions feature within helical chains parallel to the *c*-axis direction. The chains are connected by methyl­ene- and cyclo­penta­dienyl-C—H⋯O(cyclo­penta­dien­yl) inter­actions.

## Graphical abstract

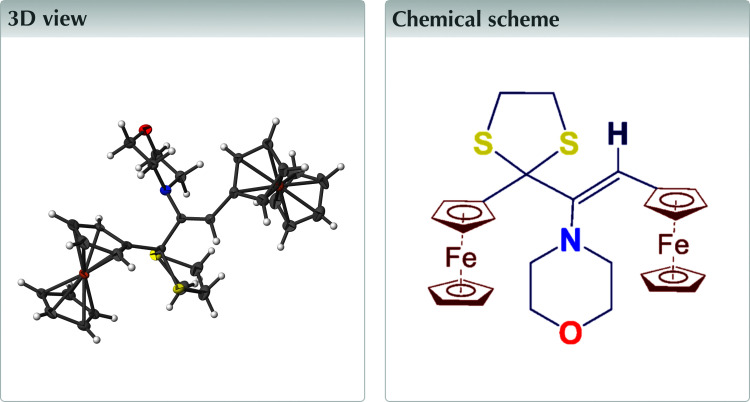

## Structure description

1,3-Di­thiol­anes, also known as *S*,*S*-thio­acetals, are stable under acidic and basic conditions (Kocieneski *et al.*, 1994 ▸). They are of importance in their applications in synthetic, organic and medicinal chemistry (Wuts Peter, 2014 ▸) and are used in synthesis as a carbonyl protecting group and for the formation of carbon–carbon bonds through metalation (Gröbel & Seebach, 1977 ▸).

The di­thiol­ane fragment is found in anti­biotics and anti­fungals such as luliconazole, which has activity against *Candida albicans*, *Malassezia spp.* and *Aspergillus fumigatus* (Khanna & Bharti, 2014 ▸).

The lipophilic character of ferrocene makes it capable of penetrating cell membranes (Ludwig *et al.*, 2019 ▸). Therefore, its incorporation into biological mol­ecules represents a matter of great inter­est in drug development. It has been pointed out that the addition of ferrocene residues in biologically active mol­ecules offers the possibility of improving the efficacy of therapeutic drugs (Patra *et al.*, 2017 ▸). In this connection, diferrocenyl-1,3-di­thiol­ane derivatives have pharmacological activity and may be considered as lead candidates for the development of new drugs or as building blocks for new mol­ecules (Mlostoń *et al.*, 2018 ▸).

**Figure 1:**
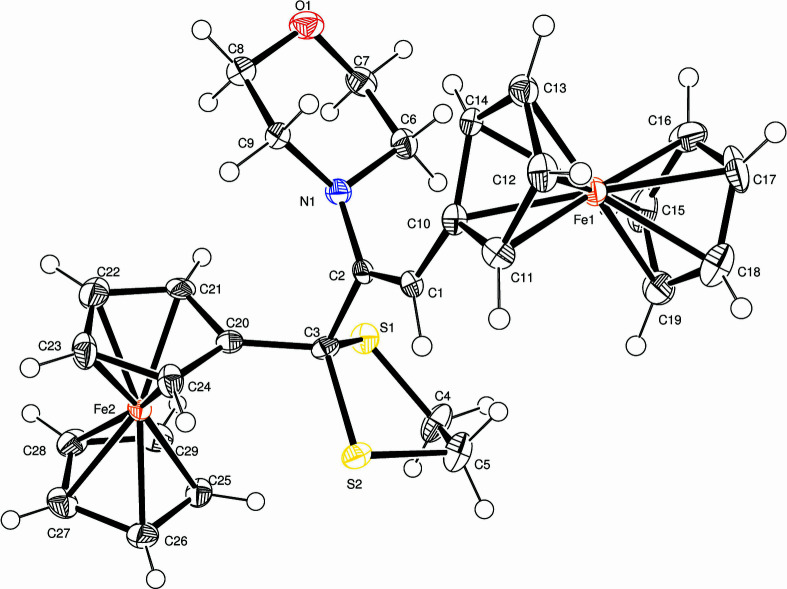
Mol­ecular structure of the title compound showing the atom-numbering scheme and displacement ellipsoids for non-H atoms at the 50% probability level.

The asymmetric unit of the title compound, Fig. 1 ▸, is formed by ferrocenyl vinyl, morpholinyl, ferrocenyl and di­thiol­ate groups. About the C1=C2 vinyl group, the morpholine fragment is *cis* to the vinyl-bound ferrocenyl residue and *trans* to the vinyl-H atom. The five-membered di­thiol­ate ring has a twisted conformation at the S1—C3 bond with puckering parameters: *q*_2_ = 0.590 (18) Å and φ_2_ = 14.0 (15)°, and asymmetry parameters (Duax *et al.*, 1976 ▸): Δ = 343.7 (24), τ = 49.7 (8), ΔCs(S1) = 61.8 (5), ΔCs(C3) = 49.5 (5) and ΔC2 (S1—C3) = 76.6 (6)°, with bond lengths of 1.858 (5) Å for S1—C3 and C4—C5 of 1.521 (11) Å. On the other hand, the six-membered morpholinyl ring, formed by the O1–C7–C6–N1–C9–C8 atoms, has a chair conformation with puckering parameters (Cremer & Pople, 1975 ▸): *Q* = 0.559 (6) Å, θ = 1.6 (5)° and φ = 24 (30)° for the calculation starting from the O1 atom through to the C8 atom, and asymmetry parameters: ΔC_2_ (O1–C7) = 1.4 (6), ΔC_2_ (C6–C7) = 3.3 (6), ΔC_2_ (C6–N1) = 2.3 (6), ΔC_2_ (O1–C8) = 2.3 (6), ΔC_s_(O1) = 0.5 (5), ΔCs(C7) = 2.3 (5), ΔCs(C6) = 2.6 (5) and ΔCs(N1) = 0.5 (5)° with an average endocyclic torsion angle of 56.7 (2)°. The orientations of the five-membered rings about the Fe1 and Fe2 atoms are very close to staggered and eclipsed, respectively.

**Figure 2:**
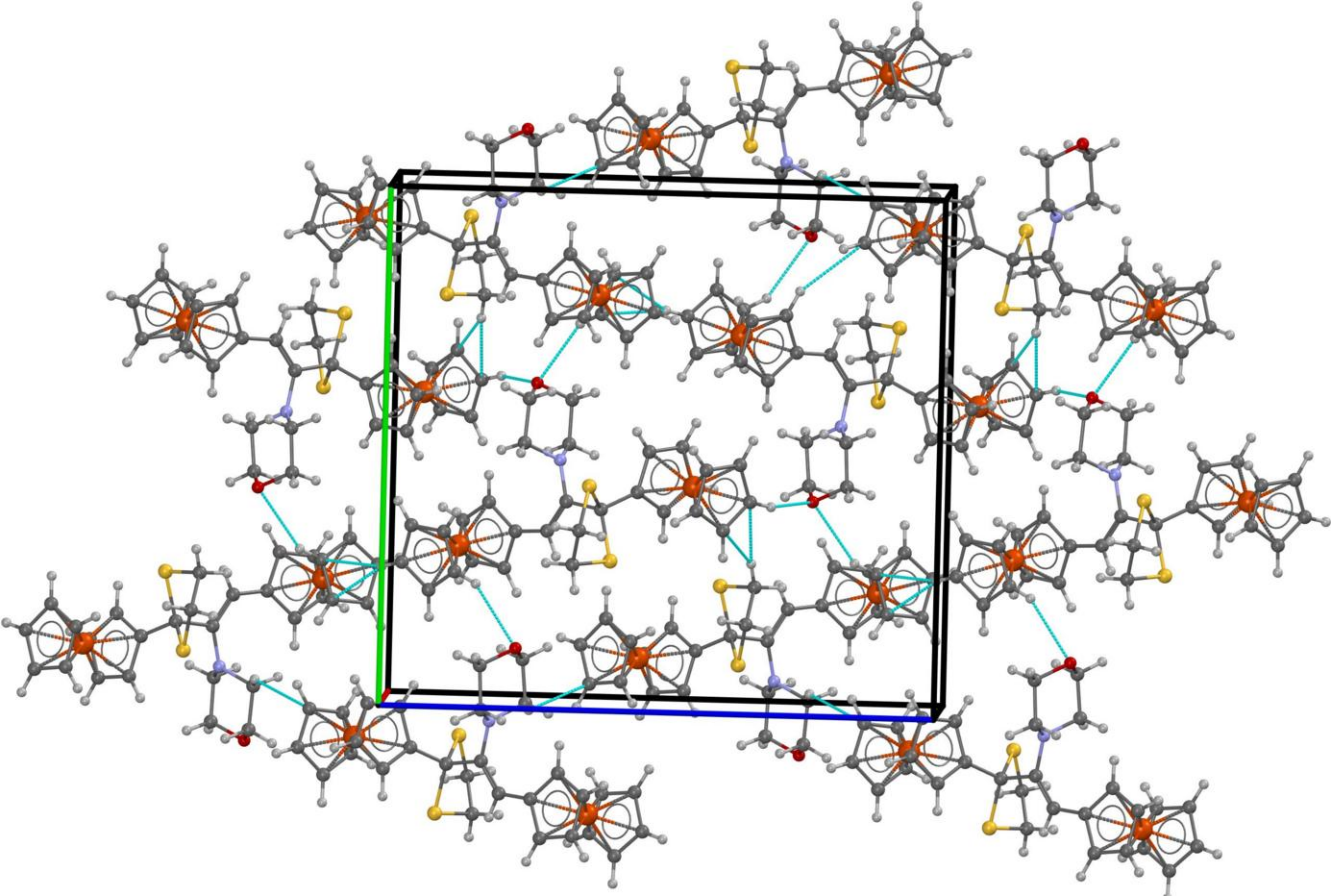
The crystal array of the title compound showing inter­molecular contacts of the type C—H⋯O and C—H⋯π inter­actions.

In the crystal, Fig. 2 ▸, cyclo­penta­dienyl-C—H⋯O(morph­olin­yl) inter­actions (Table 1 ▸)feature within helical chains parallel to the *c*-axis direction. The chains are connected within a three-dimensional architecture *via* methyl­ene- and cyclo­penta­dienyl-C—H⋯O(cyclo­penta­dien­yl) inter­actions.

## Synthesis and crystallization

**Figure 3:**
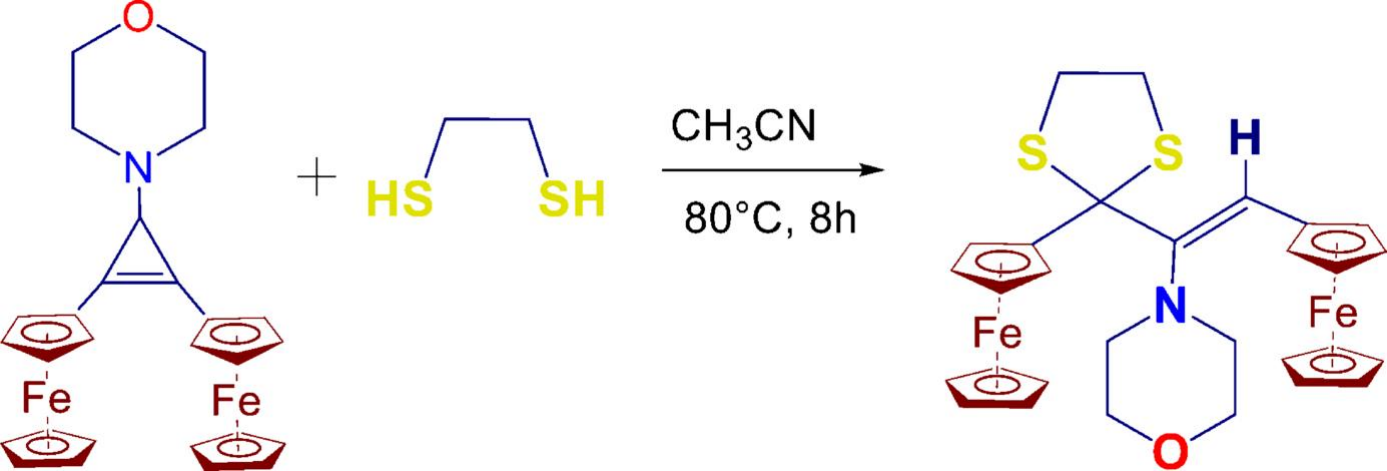
Reaction scheme.

1,2-Ethano­dithiol (15 mmol) was added to a solution of 1-morpholino-2,3-diferrocenyl­cyclo­propenonylium tetra­fluorido­borate (10 mmol) in aceto­nitrile (30 ml), and the mixture was stirred in a dry inert atmosphere under reflux for 8 h. The solvents were removed *in vacuo*, and the residues underwent chromatography on alumina (hexa­ne–di­chloro­methane, 4:1 *v*:*v*). Suitable orange crystals of 2-ferrocenyl-2-[(2-ferrocenylethen­yl)(morpholin-4-yl)meth­yl]-1,3-di­thio­l­ane were obtained by the slow evaporation of its saturated di­chloro­methane/hexane (ratio 1:2 *v*/*v*) solution. Yield (25%), m.p. 460–461 K. The reaction scheme is shown in Fig. 3 ▸.

^1^H NMR (400 MHz, CDCl_3_) δ: 274–2.76 (4*H*, *t*, 2NCH_2_, 4 Hz), 3.29–3.35 (2*H*, *m*, SCH_2_), 3.48–3.52 (2*H*, *m*, SCH_2_), 3.55–3.26 (4*H*, *t*, 2OCH_2_, 4 Hz), 4.10 (5*H*, *s*, C_5_H_5_), 4.29 (5*H*, *s*, C_5_H_5_), 4.13 (2*H*, *m*, C_5_H_4_), 4.20 (2*H*, *m*, C_5_H_4_), 4.34 (2*H*, *m*, C_5_H_4_), 4.35 (2*H*, *m*, C_5_H_4_), 6.96 (1*H*, *s*, =CH) p.p.m.., ^13^C NMR (100 MHz, CDCl_3_) δ: 39.48 (SCH_2_), 51.16 (NCH_2_), 67.15 (OCH_2_), 69.24 (SCS), 69.13, 69.74 (C_5_H_5_), 67.61, 68.71, 69.93, 70.29 (2 C_5_H_4_), 81.24, 95.89 (C_*ipso*_ Fc), 119.84 (2 C), 149.13 (–C=) p.p.m., MS: *m*/*z* 585 [*M*]^+^. Analysis calculated for C_29_H_31_Fe_2_NOS_2_: C, 59.51, H, 5.34, N, 2.39%. Found C, 60.05, H, 5.41, N, 2.45%.

## Refinement

Crystal data, data collection and structure refinement details are summarized in Table 2 ▸. The atoms of the methyl­ene-C4 group are disordered over two sets of sites and were refined with equivalent anisotropic displacement parameters to yield occupancies of 0.782 (13):0.218 (13).

## Supplementary Material

Crystal structure: contains datablock(s) I, global. DOI: 10.1107/S2414314624002347/tk4101sup1.cif

Structure factors: contains datablock(s) I. DOI: 10.1107/S2414314624002347/tk4101Isup2.hkl

CCDC reference: 2339505

Additional supporting information:  crystallographic information; 3D view; checkCIF report

## Figures and Tables

**Table 1:** Hydrogen-bond geometry (Å, °) *Cg*1–*Cg*3 are the centroids of the (C10–C14), (C25–C29) and (C15–C19) rings, respectively.

*D*—H⋯*A*	*D*—H	H⋯*A*	*D*⋯*A*	*D*—H⋯*A*
C27—H27⋯O1^i^	0.95	2.59	3.492 (7)	158
C4—H4*B*⋯*Cg*1^ii^	0.95	2.89	3.796 (9)	153
C7—H7*A*⋯*Cg*2^iii^	0.95	2.92	3.733 (6)	140
C17—H17⋯*Cg*3^iv^	0.95	2.74	3.623 (7)	155

Symmetry codes: (i) 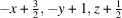 ; (ii) 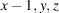 ; (iii) 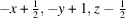 ; (iv) 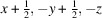 .

**Table 2:** Experimental details

Crystal data	
Chemical formula	[Fe_2_(C_5_H_5_)_2_(C_19_H_21_NOS_2_)]
*M*_r_	585.37
Crystal system, space group	Orthorhombic, *P*2_1_2_1_2_1_
Temperature (K)	130
*a*, *b*, *c* (Å)	7.5425 (3), 17.6838 (9), 18.8920 (11)
*V* (Å^3^)	2519.8 (2)
*Z*	4
Radiation type	Mo *K*α
μ (mm^−1^)	1.34
Crystal size (mm)	0.54 × 0.44 × 0.12

Data collection	
Diffractometer	Xcalibur, Atlas, Gemini
Absorption correction	Analytical (*CrysAlis RED*; Agilent, 2013 ▸)
*T*_min_, *T*_max_	0.022, 0.250
No. of measured, independent and observed [*I* > 2σ(*I*)] reflections	9130, 5847, 5231
*R*_int_	0.045
(sin θ/λ)_max_ (Å^−1^)	0.705

Refinement	
*R*[*F* ^2^ > 2σ(*F* ^2^)], *wR*(*F* ^2^), *S*	0.056, 0.139, 1.06
No. of reflections	5847
No. of parameters	320
H-atom treatment	H-atom parameters constrained
Δρ_max_, Δρ_min_ (e Å^−3^)	1.23, −1.23
Absolute structure	Flack *x* determined using 1778 quotients [(*I* ^+^)−(*I* ^−^)]/[(*I* ^+^)+(*I* ^−^)] (Parsons *et al.*, 2013 ▸)
Absolute structure parameter	0.027 (18)

Computer programs: *CrysAlis PRO* and *CrysAlis RED* (Agilent, 2013 ▸), *SHELXT2018* (Sheldrick, 2015*a* ▸), *SHELXL2018* (Sheldrick, 2015*b* ▸), *ORTEP-3 for Windows* (Farrugia, 2012 ▸) and *Mercury* (Macrae *et al.*, 2020 ▸).

## full crystallographic data

### Crystal data

**Table:**

[Fe_2_(C_5_H_5_)_2_(C_19_H_21_NOS_2_)]	*D*_x_ = 1.543 Mg m^−^^3^
*M**_r_* = 585.37	Mo *K*α radiation, λ = 0.71073 Å
Orthorhombic, *P*2_1_2_1_2_1_	Cell parameters from 3368 reflections
*a* = 7.5425 (3) Å	θ = 5.2–29.4°
*b* = 17.6838 (9) Å	µ = 1.34 mm^−^^1^
*c* = 18.8920 (11) Å	*T* = 130 K
*V* = 2519.8 (2) Å^3^	Prism, brown
*Z* = 4	0.54 × 0.44 × 0.12 mm
*F*(000) = 1216	

### Data collection

**Table:**

Xcalibur, Atlas, Gemini diffractometer	5847 independent reflections
Graphite monochromator	5231 reflections with *I* > 2σ(*I*)
Detector resolution: 10.4685 pixels mm^-1^	*R*_int_ = 0.045
ω scans	θ_max_ = 30.1°, θ_min_ = 3.5°
Absorption correction: analytical (*CrysAlis RED*; Agilent, 2013)	*h* = −6→10
*T*_min_ = 0.022, *T*_max_ = 0.250	*k* = −15→24
9130 measured reflections	*l* = −17→26

### Refinement

**Table:**

Refinement on *F*^2^	Secondary atom site location: difference Fourier map
Least-squares matrix: full	Hydrogen site location: inferred from neighbouring sites
*R*[*F*^2^ > 2σ(*F*^2^)] = 0.056	H-atom parameters constrained
*wR*(*F*^2^) = 0.139	*w* = 1/[σ^2^(*F*_o_^2^) + (0.0712*P*)^2^ + 0.1471*P*] where *P* = (*F*_o_^2^ + 2*F*_c_^2^)/3
*S* = 1.06	(Δ/σ)_max_ < 0.001
5847 reflections	Δρ_max_ = 1.23 e Å^−^^3^
320 parameters	Δρ_min_ = −1.23 e Å^−^^3^
0 restraints	Absolute structure: Flack *x* determined using 1778 quotients [(*I*^+^)-(*I*^-^)]/[(*I*^+^)+(*I*^-^)] (Parsons *et al.*, 2013)
Primary atom site location: structure-invariant direct methods	Absolute structure parameter: 0.027 (18)

### Special details

**Table:**

Geometry. All esds (except the esd in the dihedral angle between two l.s. planes) are estimated using the full covariance matrix. The cell esds are taken into account individually in the estimation of esds in distances, angles and torsion angles; correlations between esds in cell parameters are only used when they are defined by crystal symmetry. An approximate (isotropic) treatment of cell esds is used for estimating esds involving l.s. planes.

### Fractional atomic coordinates and isotropic or equivalent isotropic displacement parameters (Å^2^)

**Table:**

	*x*	*y*	*z*	*U*_iso_*/*U*_eq_	Occ. (<1)
C1	0.6462 (7)	0.3262 (3)	0.2856 (3)	0.0184 (11)	
H1	0.606180	0.278710	0.302928	0.022*	
C2	0.5778 (7)	0.3857 (3)	0.3200 (3)	0.0164 (11)	
C3	0.4556 (7)	0.3753 (3)	0.3847 (3)	0.0176 (10)	
C4	0.1034 (10)	0.3431 (5)	0.3520 (5)	0.034 (2)	0.782 (13)
H4A	0.026843	0.334821	0.393800	0.041*	0.782 (13)
H4B	0.026165	0.351702	0.310356	0.041*	0.782 (13)
C4P	0.177 (4)	0.3480 (18)	0.3075 (19)	0.034 (2)	0.218 (13)
H4PA	0.047803	0.351224	0.298393	0.041*	0.218 (13)
H4PB	0.239322	0.353020	0.261684	0.041*	0.218 (13)
C5	0.2191 (8)	0.2740 (4)	0.3395 (4)	0.0338 (15)	
H5A	0.268795	0.274712	0.291039	0.041*	
H5B	0.148982	0.227075	0.345535	0.041*	
C6	0.5293 (8)	0.4979 (3)	0.2434 (3)	0.0244 (12)	
H6A	0.404142	0.481627	0.239108	0.029*	
H6B	0.593904	0.481116	0.200573	0.029*	
C7	0.5391 (8)	0.5828 (3)	0.2501 (3)	0.0268 (12)	
H7A	0.490242	0.606267	0.206710	0.032*	
H7B	0.465229	0.599228	0.290606	0.032*	
C8	0.7914 (8)	0.5758 (3)	0.3227 (3)	0.0264 (12)	
H8A	0.722502	0.592520	0.364499	0.032*	
H8B	0.914846	0.593825	0.328617	0.032*	
C9	0.7905 (7)	0.4905 (3)	0.3192 (3)	0.0208 (11)	
H9A	0.868833	0.473088	0.280452	0.025*	
H9B	0.835530	0.469225	0.364270	0.025*	
C10	0.7709 (7)	0.3204 (3)	0.2263 (3)	0.0193 (11)	
C11	0.8416 (7)	0.2473 (3)	0.2068 (3)	0.0223 (12)	
H11	0.817860	0.200934	0.230297	0.027*	
C12	0.9524 (7)	0.2565 (3)	0.1467 (3)	0.0235 (12)	
H12	1.015044	0.217531	0.122817	0.028*	
C13	0.9528 (7)	0.3343 (3)	0.1286 (3)	0.0220 (11)	
H13	1.017439	0.356530	0.090791	0.026*	
C14	0.8403 (7)	0.3734 (3)	0.1765 (3)	0.0188 (11)	
H14	0.815352	0.426084	0.175621	0.023*	
C15	0.4455 (8)	0.3252 (4)	0.1031 (4)	0.0330 (15)	
H15	0.382220	0.364764	0.125998	0.040*	
C16	0.5535 (9)	0.3328 (4)	0.0423 (4)	0.0393 (18)	
H16	0.575569	0.378270	0.017001	0.047*	
C17	0.6235 (8)	0.2596 (5)	0.0257 (3)	0.0396 (18)	
H17	0.701159	0.247630	−0.012305	0.048*	
C18	0.5559 (8)	0.2092 (4)	0.0760 (4)	0.0352 (15)	
H18	0.579266	0.156479	0.077488	0.042*	
C19	0.4484 (8)	0.2488 (4)	0.1238 (4)	0.0312 (14)	
H19	0.387975	0.227760	0.163245	0.037*	
C20	0.5469 (6)	0.4080 (3)	0.4483 (3)	0.0186 (11)	
C21	0.5531 (8)	0.4860 (3)	0.4679 (3)	0.0233 (12)	
H21	0.488755	0.525816	0.445987	0.028*	
C22	0.6713 (8)	0.4938 (4)	0.5256 (3)	0.0330 (15)	
H22	0.700553	0.539908	0.548606	0.040*	
C23	0.7387 (7)	0.4214 (4)	0.5433 (3)	0.0291 (13)	
H23	0.819272	0.410200	0.580494	0.035*	
C24	0.6634 (7)	0.3687 (4)	0.4949 (3)	0.0228 (12)	
H24	0.686980	0.315958	0.493921	0.027*	
C25	0.2141 (7)	0.3841 (3)	0.5604 (3)	0.0240 (12)	
H25	0.145269	0.362048	0.523752	0.029*	
C26	0.3266 (8)	0.3445 (4)	0.6076 (3)	0.0275 (13)	
H26	0.345859	0.291407	0.608294	0.033*	
C27	0.4053 (8)	0.3984 (4)	0.6536 (3)	0.0285 (13)	
H27	0.487580	0.387627	0.690309	0.034*	
C28	0.3397 (8)	0.4713 (4)	0.6352 (3)	0.0276 (13)	
H28	0.369460	0.517597	0.657731	0.033*	
C29	0.2213 (8)	0.4622 (4)	0.5770 (3)	0.0291 (13)	
H29	0.158548	0.501472	0.553572	0.035*	
Fe1	0.69918 (10)	0.29194 (4)	0.12507 (4)	0.01799 (19)	
Fe2	0.46821 (10)	0.42310 (5)	0.55104 (4)	0.01902 (19)	
N1	0.6094 (6)	0.4643 (3)	0.3067 (2)	0.0194 (9)	
O1	0.7173 (6)	0.6085 (2)	0.2605 (2)	0.0296 (10)	
S1	0.24352 (16)	0.42501 (9)	0.36629 (8)	0.0240 (3)	
S2	0.39473 (18)	0.27808 (8)	0.40423 (8)	0.0226 (3)	

### Atomic displacement parameters (Å^2^)

**Table:**

	*U*^11^	*U*^22^	*U*^33^	*U*^12^	*U*^13^	*U*^23^
C1	0.021 (2)	0.020 (3)	0.014 (2)	−0.002 (2)	−0.004 (2)	0.0020 (19)
C2	0.016 (2)	0.020 (3)	0.013 (2)	−0.002 (2)	−0.004 (2)	0.0024 (19)
C3	0.016 (2)	0.011 (2)	0.025 (3)	0.001 (2)	−0.001 (2)	0.0024 (19)
C4	0.017 (3)	0.036 (4)	0.050 (6)	−0.005 (3)	−0.006 (3)	−0.012 (4)
C4P	0.017 (3)	0.036 (4)	0.050 (6)	−0.005 (3)	−0.006 (3)	−0.012 (4)
C5	0.028 (3)	0.034 (4)	0.039 (4)	−0.002 (3)	−0.008 (3)	−0.011 (3)
C6	0.024 (3)	0.028 (3)	0.021 (3)	0.001 (3)	−0.003 (2)	0.000 (2)
C7	0.031 (3)	0.026 (3)	0.024 (3)	0.004 (3)	0.002 (3)	0.003 (2)
C8	0.027 (3)	0.027 (3)	0.025 (3)	−0.005 (3)	0.000 (3)	0.000 (2)
C9	0.021 (2)	0.023 (3)	0.019 (3)	−0.005 (2)	−0.001 (2)	0.000 (2)
C10	0.019 (2)	0.021 (3)	0.018 (3)	0.001 (2)	−0.003 (2)	−0.003 (2)
C11	0.021 (3)	0.024 (3)	0.022 (3)	0.003 (2)	0.001 (2)	0.002 (2)
C12	0.020 (2)	0.027 (3)	0.023 (3)	0.006 (2)	−0.001 (2)	−0.006 (2)
C13	0.019 (2)	0.027 (3)	0.019 (3)	−0.001 (2)	0.001 (2)	−0.002 (2)
C14	0.020 (3)	0.020 (3)	0.016 (2)	−0.004 (2)	−0.003 (2)	−0.0015 (19)
C15	0.021 (3)	0.040 (4)	0.038 (4)	0.004 (3)	−0.012 (3)	−0.011 (3)
C16	0.039 (4)	0.041 (4)	0.037 (4)	−0.020 (3)	−0.022 (3)	0.020 (3)
C17	0.023 (3)	0.080 (6)	0.016 (3)	−0.009 (3)	0.000 (3)	−0.014 (3)
C18	0.030 (3)	0.031 (4)	0.044 (4)	−0.003 (3)	−0.009 (3)	−0.011 (3)
C19	0.020 (2)	0.046 (4)	0.027 (3)	−0.011 (3)	−0.005 (3)	0.002 (3)
C20	0.014 (2)	0.023 (3)	0.018 (2)	−0.004 (2)	0.005 (2)	0.001 (2)
C21	0.027 (3)	0.026 (3)	0.017 (3)	−0.008 (2)	0.011 (2)	0.000 (2)
C22	0.028 (3)	0.046 (4)	0.025 (3)	−0.015 (3)	0.009 (3)	−0.008 (3)
C23	0.018 (2)	0.051 (4)	0.019 (3)	−0.005 (3)	−0.001 (2)	−0.006 (3)
C24	0.015 (2)	0.033 (3)	0.020 (3)	0.001 (2)	−0.001 (2)	−0.004 (2)
C25	0.014 (2)	0.036 (3)	0.022 (3)	−0.006 (2)	0.005 (2)	−0.004 (2)
C26	0.027 (3)	0.029 (3)	0.026 (3)	−0.006 (3)	0.006 (3)	0.003 (2)
C27	0.027 (3)	0.040 (4)	0.019 (3)	−0.001 (3)	0.008 (2)	0.000 (2)
C28	0.028 (3)	0.029 (3)	0.026 (3)	−0.007 (2)	0.012 (3)	−0.007 (2)
C29	0.023 (3)	0.032 (3)	0.032 (3)	0.004 (3)	0.009 (3)	0.004 (2)
Fe1	0.0182 (3)	0.0201 (4)	0.0156 (4)	−0.0006 (3)	−0.0010 (3)	−0.0012 (3)
Fe2	0.0164 (3)	0.0252 (4)	0.0155 (4)	−0.0026 (3)	0.0021 (3)	−0.0013 (3)
N1	0.017 (2)	0.022 (2)	0.019 (2)	−0.0008 (19)	−0.0028 (19)	0.0033 (18)
O1	0.036 (2)	0.021 (2)	0.032 (2)	−0.0023 (19)	0.003 (2)	0.0049 (17)
S1	0.0162 (5)	0.0282 (8)	0.0276 (7)	0.0028 (5)	−0.0020 (5)	−0.0003 (6)
S2	0.0217 (6)	0.0198 (7)	0.0261 (7)	−0.0040 (6)	0.0027 (6)	−0.0003 (5)

### Geometric parameters (Å, º)

**Table:**

C1—C2	1.340 (8)	C14—H14	0.9500
C1—C10	1.467 (8)	C15—C19	1.406 (10)
C1—H1	0.9500	C15—C16	1.415 (10)
C2—N1	1.433 (7)	C15—Fe1	2.045 (6)
C2—C3	1.541 (7)	C15—H15	0.9500
C3—C20	1.502 (7)	C16—C17	1.432 (11)
C3—S2	1.817 (5)	C16—Fe1	2.043 (6)
C3—S1	1.858 (5)	C16—H16	0.9500
C4—C5	1.521 (11)	C17—C18	1.399 (10)
C4—S1	1.813 (8)	C17—Fe1	2.044 (6)
C4—H4A	0.9900	C17—H17	0.9500
C4—H4B	0.9900	C18—C19	1.402 (9)
C4P—C5	1.48 (3)	C18—Fe1	2.041 (6)
C4P—S1	1.83 (3)	C18—H18	0.9500
C4P—H4PA	0.9900	C19—Fe1	2.040 (6)
C4P—H4PB	0.9900	C19—H19	0.9500
C5—S2	1.804 (6)	C20—C24	1.424 (8)
C5—H5A	0.9900	C20—C21	1.430 (8)
C5—H5B	0.9900	C20—Fe2	2.046 (5)
C6—N1	1.466 (7)	C21—C22	1.414 (9)
C6—C7	1.509 (9)	C21—Fe2	2.028 (5)
C6—H6A	0.9900	C21—H21	0.9500
C6—H6B	0.9900	C22—C23	1.418 (10)
C7—O1	1.433 (8)	C22—Fe2	2.035 (6)
C7—H7A	0.9900	C22—H22	0.9500
C7—H7B	0.9900	C23—C24	1.423 (8)
C8—O1	1.425 (7)	C23—Fe2	2.046 (5)
C8—C9	1.511 (8)	C23—H23	0.9500
C8—H8A	0.9900	C24—Fe2	2.053 (6)
C8—H8B	0.9900	C24—H24	0.9500
C9—N1	1.461 (7)	C25—C26	1.416 (9)
C9—H9A	0.9900	C25—C29	1.417 (8)
C9—H9B	0.9900	C25—Fe2	2.044 (5)
C10—C14	1.427 (7)	C25—H25	0.9500
C10—C11	1.445 (8)	C26—C27	1.420 (9)
C10—Fe1	2.051 (5)	C26—Fe2	2.053 (6)
C11—C12	1.420 (8)	C26—H26	0.9500
C11—Fe1	2.040 (6)	C27—C28	1.423 (9)
C11—H11	0.9500	C27—Fe2	2.042 (6)
C12—C13	1.417 (8)	C27—H27	0.9500
C12—Fe1	2.051 (6)	C28—C29	1.426 (9)
C12—H12	0.9500	C28—Fe2	2.048 (6)
C13—C14	1.421 (8)	C28—H28	0.9500
C13—Fe1	2.055 (5)	C29—Fe2	2.046 (6)
C13—H13	0.9500	C29—H29	0.9500
C14—Fe1	2.038 (5)		
			
C2—C1—C10	132.2 (5)	C22—C23—H23	126.4
C2—C1—H1	113.9	C24—C23—H23	126.4
C10—C1—H1	113.9	Fe2—C23—H23	126.0
C1—C2—N1	127.8 (5)	C23—C24—C20	108.9 (5)
C1—C2—C3	121.3 (5)	C23—C24—Fe2	69.4 (3)
N1—C2—C3	110.8 (4)	C20—C24—Fe2	69.4 (3)
C20—C3—C2	108.3 (4)	C23—C24—H24	125.6
C20—C3—S2	108.5 (4)	C20—C24—H24	125.6
C2—C3—S2	115.2 (4)	Fe2—C24—H24	127.2
C20—C3—S1	111.2 (4)	C26—C25—C29	108.7 (5)
C2—C3—S1	108.1 (3)	C26—C25—Fe2	70.1 (3)
S2—C3—S1	105.6 (3)	C29—C25—Fe2	69.8 (3)
C5—C4—S1	109.3 (5)	C26—C25—H25	125.7
C5—C4—H4A	109.8	C29—C25—H25	125.7
S1—C4—H4A	109.8	Fe2—C25—H25	126.0
C5—C4—H4B	109.8	C25—C26—C27	107.7 (6)
S1—C4—H4B	109.8	C25—C26—Fe2	69.5 (3)
H4A—C4—H4B	108.3	C27—C26—Fe2	69.3 (3)
C5—C4P—S1	110.6 (19)	C25—C26—H26	126.2
C5—C4P—H4PA	109.5	C27—C26—H26	126.2
S1—C4P—H4PA	109.5	Fe2—C26—H26	126.6
C5—C4P—H4PB	109.5	C26—C27—C28	108.2 (5)
S1—C4P—H4PB	109.5	C26—C27—Fe2	70.1 (3)
H4PA—C4P—H4PB	108.1	C28—C27—Fe2	69.9 (3)
C4P—C5—S2	113.6 (12)	C26—C27—H27	125.9
C4—C5—S2	106.5 (5)	C28—C27—H27	125.9
C4—C5—H5A	110.4	Fe2—C27—H27	125.7
S2—C5—H5A	110.4	C27—C28—C29	107.7 (5)
C4—C5—H5B	110.4	C27—C28—Fe2	69.4 (3)
S2—C5—H5B	110.4	C29—C28—Fe2	69.6 (3)
H5A—C5—H5B	108.6	C27—C28—H28	126.2
N1—C6—C7	108.3 (5)	C29—C28—H28	126.2
N1—C6—H6A	110.0	Fe2—C28—H28	126.5
C7—C6—H6A	110.0	C25—C29—C28	107.7 (5)
N1—C6—H6B	110.0	C25—C29—Fe2	69.7 (3)
C7—C6—H6B	110.0	C28—C29—Fe2	69.7 (3)
H6A—C6—H6B	108.4	C25—C29—H29	126.2
O1—C7—C6	111.9 (5)	C28—C29—H29	126.2
O1—C7—H7A	109.2	Fe2—C29—H29	126.1
C6—C7—H7A	109.2	C14—Fe1—C19	138.9 (3)
O1—C7—H7B	109.2	C14—Fe1—C11	68.7 (2)
C6—C7—H7B	109.2	C19—Fe1—C11	110.7 (2)
H7A—C7—H7B	107.9	C14—Fe1—C18	178.5 (3)
O1—C8—C9	111.6 (5)	C19—Fe1—C18	40.2 (3)
O1—C8—H8A	109.3	C11—Fe1—C18	110.2 (3)
C9—C8—H8A	109.3	C14—Fe1—C16	113.3 (3)
O1—C8—H8B	109.3	C19—Fe1—C16	67.9 (3)
C9—C8—H8B	109.3	C11—Fe1—C16	177.9 (3)
H8A—C8—H8B	108.0	C18—Fe1—C16	67.7 (3)
N1—C9—C8	109.1 (5)	C14—Fe1—C17	141.4 (3)
N1—C9—H9A	109.9	C19—Fe1—C17	68.0 (3)
C8—C9—H9A	109.9	C11—Fe1—C17	137.3 (3)
N1—C9—H9B	109.9	C18—Fe1—C17	40.1 (3)
C8—C9—H9B	109.9	C16—Fe1—C17	41.0 (3)
H9A—C9—H9B	108.3	C14—Fe1—C15	112.4 (3)
C14—C10—C11	106.5 (5)	C19—Fe1—C15	40.3 (3)
C14—C10—C1	133.8 (5)	C11—Fe1—C15	139.2 (3)
C11—C10—C1	119.6 (5)	C18—Fe1—C15	67.6 (3)
C14—C10—Fe1	69.1 (3)	C16—Fe1—C15	40.5 (3)
C11—C10—Fe1	68.9 (3)	C17—Fe1—C15	68.5 (3)
C1—C10—Fe1	124.1 (4)	C14—Fe1—C10	40.9 (2)
C12—C11—C10	108.6 (5)	C19—Fe1—C10	110.3 (2)
C12—C11—Fe1	70.1 (3)	C11—Fe1—C10	41.4 (2)
C10—C11—Fe1	69.7 (3)	C18—Fe1—C10	137.7 (3)
C12—C11—H11	125.7	C16—Fe1—C10	140.3 (3)
C10—C11—H11	125.7	C17—Fe1—C10	177.7 (3)
Fe1—C11—H11	126.1	C15—Fe1—C10	111.4 (2)
C13—C12—C11	107.8 (5)	C14—Fe1—C12	68.6 (2)
C13—C12—Fe1	70.0 (3)	C19—Fe1—C12	138.8 (3)
C11—C12—Fe1	69.3 (3)	C11—Fe1—C12	40.6 (2)
C13—C12—H12	126.1	C18—Fe1—C12	111.4 (3)
C11—C12—H12	126.1	C16—Fe1—C12	139.6 (3)
Fe1—C12—H12	126.2	C17—Fe1—C12	110.9 (3)
C12—C13—C14	108.5 (5)	C15—Fe1—C12	179.0 (3)
C12—C13—Fe1	69.6 (3)	C10—Fe1—C12	69.1 (2)
C14—C13—Fe1	69.0 (3)	C14—Fe1—C13	40.6 (2)
C12—C13—H13	125.7	C19—Fe1—C13	178.7 (3)
C14—C13—H13	125.7	C11—Fe1—C13	68.1 (2)
Fe1—C13—H13	127.2	C18—Fe1—C13	140.2 (2)
C13—C14—C10	108.6 (5)	C16—Fe1—C13	113.4 (2)
C13—C14—Fe1	70.3 (3)	C17—Fe1—C13	113.0 (2)
C10—C14—Fe1	70.0 (3)	C15—Fe1—C13	140.6 (3)
C13—C14—H14	125.7	C10—Fe1—C13	68.6 (2)
C10—C14—H14	125.7	C12—Fe1—C13	40.4 (2)
Fe1—C14—H14	125.5	C21—Fe2—C22	40.7 (2)
C19—C15—C16	107.9 (6)	C21—Fe2—C27	157.7 (2)
C19—C15—Fe1	69.7 (4)	C22—Fe2—C27	122.1 (3)
C16—C15—Fe1	69.7 (4)	C21—Fe2—C25	123.2 (2)
C19—C15—H15	126.1	C22—Fe2—C25	158.9 (3)
C16—C15—H15	126.1	C27—Fe2—C25	68.1 (2)
Fe1—C15—H15	126.1	C21—Fe2—C23	68.8 (3)
C15—C16—C17	107.8 (6)	C22—Fe2—C23	40.7 (3)
C15—C16—Fe1	69.8 (4)	C27—Fe2—C23	107.3 (3)
C17—C16—Fe1	69.5 (4)	C25—Fe2—C23	159.4 (3)
C15—C16—H16	126.1	C21—Fe2—C29	106.7 (3)
C17—C16—H16	126.1	C22—Fe2—C29	122.3 (3)
Fe1—C16—H16	126.2	C27—Fe2—C29	68.5 (3)
C18—C17—C16	107.0 (6)	C25—Fe2—C29	40.5 (2)
C18—C17—Fe1	69.9 (4)	C23—Fe2—C29	158.4 (3)
C16—C17—Fe1	69.5 (4)	C21—Fe2—C20	41.1 (2)
C18—C17—H17	126.5	C22—Fe2—C20	68.8 (2)
C16—C17—H17	126.5	C27—Fe2—C20	159.9 (2)
Fe1—C17—H17	125.7	C25—Fe2—C20	108.1 (2)
C17—C18—C19	109.3 (6)	C23—Fe2—C20	69.0 (2)
C17—C18—Fe1	70.1 (4)	C29—Fe2—C20	122.4 (2)
C19—C18—Fe1	69.9 (4)	C21—Fe2—C28	121.5 (3)
C17—C18—H18	125.4	C22—Fe2—C28	106.5 (3)
C19—C18—H18	125.4	C27—Fe2—C28	40.7 (2)
Fe1—C18—H18	126.3	C25—Fe2—C28	68.2 (2)
C18—C19—C15	108.1 (6)	C23—Fe2—C28	122.2 (2)
C18—C19—Fe1	70.0 (4)	C29—Fe2—C28	40.8 (3)
C15—C19—Fe1	70.1 (3)	C20—Fe2—C28	158.1 (2)
C18—C19—H19	126.0	C21—Fe2—C26	159.8 (2)
C15—C19—H19	126.0	C22—Fe2—C26	158.6 (3)
Fe1—C19—H19	125.6	C27—Fe2—C26	40.6 (2)
C24—C20—C21	106.9 (5)	C25—Fe2—C26	40.4 (2)
C24—C20—C3	126.2 (5)	C23—Fe2—C26	123.1 (3)
C21—C20—C3	126.4 (5)	C29—Fe2—C26	68.3 (3)
C24—C20—Fe2	69.9 (3)	C20—Fe2—C26	123.8 (2)
C21—C20—Fe2	68.8 (3)	C28—Fe2—C26	68.4 (3)
C3—C20—Fe2	132.6 (3)	C21—Fe2—C24	68.3 (2)
C22—C21—C20	108.3 (6)	C22—Fe2—C24	68.0 (3)
C22—C21—Fe2	69.9 (3)	C27—Fe2—C24	123.8 (3)
C20—C21—Fe2	70.2 (3)	C25—Fe2—C24	124.0 (2)
C22—C21—H21	125.9	C23—Fe2—C24	40.6 (2)
C20—C21—H21	125.9	C29—Fe2—C24	159.2 (2)
Fe2—C21—H21	125.7	C20—Fe2—C24	40.7 (2)
C21—C22—C23	108.7 (6)	C28—Fe2—C24	159.2 (2)
C21—C22—Fe2	69.4 (3)	C26—Fe2—C24	108.9 (3)
C23—C22—Fe2	70.1 (4)	C2—N1—C9	115.7 (4)
C21—C22—H22	125.7	C2—N1—C6	117.8 (4)
C23—C22—H22	125.7	C9—N1—C6	112.9 (4)
Fe2—C22—H22	126.4	C8—O1—C7	110.6 (4)
C22—C23—C24	107.3 (5)	C4—S1—C3	98.7 (3)
C22—C23—Fe2	69.3 (3)	C4P—S1—C3	89.9 (9)
C24—C23—Fe2	70.0 (3)	C5—S2—C3	94.9 (3)
			
C10—C1—C2—N1	0.6 (9)	C24—C20—C21—C22	−0.1 (6)
C10—C1—C2—C3	−176.2 (5)	C3—C20—C21—C22	−172.3 (5)
C1—C2—C3—C20	115.7 (5)	Fe2—C20—C21—C22	59.7 (4)
N1—C2—C3—C20	−61.7 (5)	C24—C20—C21—Fe2	−59.8 (4)
C1—C2—C3—S2	−6.0 (6)	C3—C20—C21—Fe2	128.0 (5)
N1—C2—C3—S2	176.7 (3)	C20—C21—C22—C23	−0.6 (6)
C1—C2—C3—S1	−123.8 (5)	Fe2—C21—C22—C23	59.3 (4)
N1—C2—C3—S1	58.9 (5)	C20—C21—C22—Fe2	−59.9 (4)
S1—C4P—C5—S2	−24 (2)	C21—C22—C23—C24	1.1 (6)
S1—C4—C5—S2	43.6 (7)	Fe2—C22—C23—C24	59.9 (4)
N1—C6—C7—O1	56.4 (6)	C21—C22—C23—Fe2	−58.8 (4)
O1—C8—C9—N1	−55.9 (6)	C22—C23—C24—C20	−1.2 (6)
C2—C1—C10—C14	−12.8 (10)	Fe2—C23—C24—C20	58.3 (4)
C2—C1—C10—C11	170.8 (6)	C22—C23—C24—Fe2	−59.5 (4)
C2—C1—C10—Fe1	−105.8 (6)	C21—C20—C24—C23	0.8 (6)
C14—C10—C11—C12	0.4 (6)	C3—C20—C24—C23	173.0 (5)
C1—C10—C11—C12	177.7 (5)	Fe2—C20—C24—C23	−58.3 (4)
Fe1—C10—C11—C12	59.5 (4)	C21—C20—C24—Fe2	59.1 (4)
C14—C10—C11—Fe1	−59.1 (4)	C3—C20—C24—Fe2	−128.7 (5)
C1—C10—C11—Fe1	118.1 (5)	C29—C25—C26—C27	−0.4 (7)
C10—C11—C12—C13	0.3 (6)	Fe2—C25—C26—C27	59.0 (4)
Fe1—C11—C12—C13	59.6 (4)	C29—C25—C26—Fe2	−59.3 (4)
C10—C11—C12—Fe1	−59.3 (4)	C25—C26—C27—C28	0.6 (7)
C11—C12—C13—C14	−1.0 (6)	Fe2—C26—C27—C28	59.7 (4)
Fe1—C12—C13—C14	58.2 (4)	C25—C26—C27—Fe2	−59.1 (4)
C11—C12—C13—Fe1	−59.2 (4)	C26—C27—C28—C29	−0.6 (7)
C12—C13—C14—C10	1.2 (6)	Fe2—C27—C28—C29	59.2 (4)
Fe1—C13—C14—C10	59.8 (4)	C26—C27—C28—Fe2	−59.9 (4)
C12—C13—C14—Fe1	−58.6 (4)	C26—C25—C29—C28	0.0 (7)
C11—C10—C14—C13	−1.0 (6)	Fe2—C25—C29—C28	−59.6 (4)
C1—C10—C14—C13	−177.7 (6)	C26—C25—C29—Fe2	59.5 (4)
Fe1—C10—C14—C13	−60.0 (4)	C27—C28—C29—C25	0.4 (7)
C11—C10—C14—Fe1	59.0 (4)	Fe2—C28—C29—C25	59.5 (4)
C1—C10—C14—Fe1	−117.7 (6)	C27—C28—C29—Fe2	−59.1 (4)
C19—C15—C16—C17	−0.1 (7)	C1—C2—N1—C9	−65.2 (7)
Fe1—C15—C16—C17	59.4 (4)	C3—C2—N1—C9	111.9 (5)
C19—C15—C16—Fe1	−59.4 (4)	C1—C2—N1—C6	72.9 (7)
C15—C16—C17—C18	0.5 (7)	C3—C2—N1—C6	−110.0 (5)
Fe1—C16—C17—C18	60.1 (4)	C8—C9—N1—C2	−164.5 (5)
C15—C16—C17—Fe1	−59.5 (4)	C8—C9—N1—C6	55.4 (6)
C16—C17—C18—C19	−0.8 (7)	C7—C6—N1—C2	165.4 (5)
Fe1—C17—C18—C19	59.0 (4)	C7—C6—N1—C9	−55.4 (6)
C16—C17—C18—Fe1	−59.8 (4)	C9—C8—O1—C7	58.4 (6)
C17—C18—C19—C15	0.8 (7)	C6—C7—O1—C8	−59.0 (6)
Fe1—C18—C19—C15	59.9 (4)	C5—C4—S1—C3	−16.1 (7)
C17—C18—C19—Fe1	−59.1 (4)	C5—C4P—S1—C3	43.3 (18)
C16—C15—C19—C18	−0.4 (7)	C20—C3—S1—C4	−134.0 (5)
Fe1—C15—C19—C18	−59.9 (4)	C2—C3—S1—C4	107.3 (5)
C16—C15—C19—Fe1	59.4 (4)	S2—C3—S1—C4	−16.5 (4)
C2—C3—C20—C24	−89.6 (6)	C20—C3—S1—C4P	−165.0 (12)
S2—C3—C20—C24	36.2 (6)	C2—C3—S1—C4P	76.2 (12)
S1—C3—C20—C24	151.8 (4)	S2—C3—S1—C4P	−47.6 (12)
C2—C3—C20—C21	81.2 (6)	C4P—C5—S2—C3	−9.1 (16)
S2—C3—C20—C21	−153.1 (5)	C4—C5—S2—C3	−50.4 (6)
S1—C3—C20—C21	−37.4 (6)	C20—C3—S2—C5	156.7 (4)
C2—C3—C20—Fe2	175.0 (4)	C2—C3—S2—C5	−81.7 (4)
S2—C3—C20—Fe2	−59.2 (6)	S1—C3—S2—C5	37.4 (3)
S1—C3—C20—Fe2	56.4 (6)		

### Hydrogen-bond geometry (Å, º)

*Cg*1–*Cg*3 are the centroids of the (C10–C14), (C25–C29) and (C15–C19) rings, respectively.

**Table:**

*D*—H···*A*	*D*—H	H···*A*	*D*···*A*	*D*—H···*A*
C27—H27···O1^i^	0.95	2.59	3.492 (7)	158
C4—H4*B*···*Cg*1^ii^	0.95	2.89	3.796 (9)	153
C7—H7*A*···*Cg*2^iii^	0.95	2.92	3.733 (6)	140
C17—H17···*Cg*3^iv^	0.95	2.74	3.623 (7)	155

Symmetry codes: (i) −*x*+3/2, −*y*+1, *z*+1/2; (ii) *x*−1, *y*, *z*; (iii) −*x*+1/2, −*y*+1, *z*−1/2; (iv) *x*+1/2, −*y*+1/2, −*z*.
